# Limonene, the compound in essential oil of nutmeg displayed antioxidant effect in sunflower oil during the deep‐frying of Chinese *Maye*

**DOI:** 10.1002/fsn3.1333

**Published:** 2019-12-11

**Authors:** Dongying Wang, Ying Dong, Qing Wang, Xuede Wang, Wenchang Fan

**Affiliations:** ^1^ College of Food Science and Technology Henan University of Technology Zhengzhou China; ^2^ Institute of Chinese Medicine Health Care Guangdong Food and Drug Vocational College Guangzhou China

**Keywords:** antioxidant effects, essential oil, limonene, nutmeg, sunflower oil

## Abstract

The deep‐frying process for plenty of fried products using vegetable oils needs safe and effective antioxidants. In the present exploration, the nutmeg essential oil (NEO) was employed as a potential antioxidant for sunflower oil during the deep‐frying of Chinese *Maye* at 180°C for 30 hr. In the comparative study, the additions for NEO at 0.12 g/kg, TBHQ at 0.12 g/kg, BHA at 0.08 g/kg, and BHT at 0.08 g/kg to sunflower oil were able to obviously improve its oxidative stability during the deep‐frying process, and their antioxidant effects were in the relative order: TBHQ at 0.12 g/kg > NEO at 0.12 g/kg > BHA at 0.08 g/kg > BHT at 0.08 g/kg (*p* < .05). Besides, NEO at 0.12 g/kg could markedly ameliorate the sensory properties including flavor, taste, crispness, and overall acceptability of the fried products, Chinese *Maye* (*p* < .05 or *p* < .01). In addition, using antioxidant activity‐guided fractionation, three active compounds including limonene, terpinolene, and geranyl acetate were isolated from NEO. Among them, limonene was demonstrated to not only significantly increase the oxidative stability of sunflower oil in the deep‐frying process, but also significantly increase the sensory properties of the fried products, Chinese *Maye* (*p* < .05 or *p* < .01). Consequently, limonene could be employed as antioxidants in sunflower oil for the deep‐frying of Chinese *Maye*, and the sunflower oil flavored by NEO could be used as frying oils for its oxidative stability and unique flavor.

## Graphical abstract

The deep‐frying process for plenty of fried products using vegetable oils needs safe antioxidants. In the present study, the nutmeg essential oil (NEO) and limonene were demonstrated to be effective in inhibiting the oxidation of sunflower oil during the deep‐frying of Chinese Maye at 180°C for 30 hr and improving the sensory properties of the fried products, Chinese Maye. Therefore, both of them could be used as antioxidants in the deep‐frying process of sunflower oil.

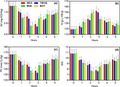

## 1 INTRODUCTION

During the deep‐frying, vegetable oils always undergo oxidative rancidity because of their inherent fatty acid compositions, which can lead to the production of free radicals and lipid degradation products and finally their oxidative deterioration (Aladedunye & Matthäus, 2014). As known, the oxidative deterioration of vegetable oils not only shortens their special shelf life, but also influences the sensory and health quality of the fried products, (Jiménez et al., 2017). Therefore, several synthetic antioxidants including *tert*‐butylhydroquinone (TBHQ), butylated hydroxyanisole (BHA), and butylated hydroxytoluene (BHT) have been employed in the frying procedure to extend the usage period of vegetable oils and improve the sensory properties of their fried products (Chirinos, Huamán, Betalleluz‐Pallardel, Pedreschi, & Campos, 2011). However, the safety of these synthetic antioxidants has long been questioned because of their negative effects on human health, such as enlargement of the liver, and transformation of ingested materials into carcinogenic substances (Hwang, Winkler‐Moser, & Liu, 2012). In the meantime, natural antioxidants (natural products/crude extracts) from spices and herbs have been used in the frying procedure, and they were considered to be safe (Wang, Liu, & Qin, 2017). For instance, the addition of a carotenoid extract from *Lycium barbarum* was able to improve the oxidative stability of extra‐virgin olive oil during a long‐term storage for 28 days at room temperature and a frying process for 180 min at 180 ± 4°C (Blasi et al., 2018; Montesano et al., 2019). Therefore, it is quite necessary and meaningful to search for natural antioxidants from herbs and spices.

As a leafy tree of height about 12 m when mature, *Myristica fragrans* Houtt. (Myristicaceae family) is important for plenty of beverages derived from its fruits and two spices used in sweet and savory cooking, mace, and nutmeg. Among them, mace is the aril, and nutmeg is the nut (Piras et al., 2012; Zhao, Wu, Wei, & Yang, 2019). Except spices, nutmeg has been used to treat nausea, anxiety, cholera, psychosis, diarrhea, flatulence, rheumatism, and stomach cramps in Chinese medicine for more than 1000 years (Acuña, Carcache, Matthew, & de Blanco, 2016). Additionally, in the continuous phytochemical investigations and pharmacological explorations, secondary metabolites including lignans, steroids, cyclobutanones, diphenylalkanes, phenylpropanediols, and essential oils have been isolated from *M. fragrans*, and natural products/crude extracts of *M. fragrans* have been demonstrated to display biological effects including antifungal, antimicrobial, anti‐inflammatory, and hepatoprotective effects (Abourashed & El‐Alfy, 2016). Above all, the antioxidant potential of *M. fragrans* has been reported by researcher all over the world. For example, the 50% acetone extract and 80% methanol extract of *M. fragrans* leaves exhibited radical‐scavenging effects against ABTS^•+^, DPPH^•^, ORAC, and HO^•^ radicals (Su et al., 2007). Interestingly, the essential oil of *M. fragrans* fruits revealed inhibitory effect against the generation for the primary and secondary oxidation products in mustard in the accelerated storage (Kapoor et al., 2013). Furthermore, the essential oil of *M. fragrans* was demonstrated to improve the oxidative and microbial stability of cooked sausage during the refrigerated storage (Šojić et al., 2015). Spontaneously, one hypothesis that nutmeg essential oil may be used as an effective natural antioxidant for the frying procedure of vegetable oils arose.

According to these considerations, the aims of the present study were to investigate the influences of nutmeg essential oil (NEO) on the oxidative stability of sunflower oil and the sensory properties of Chinese *Maye*, and search for the compounds in NEO that reveal antioxidant effect during the deep‐frying procedure.

## 2 MATERIALS AND METHODS

### 2.1 Materials and chemicals

The nuts of *M. fragrans* (nutmeg, 4 × 10.0 kg) harvested from Jinghong, China, were bought from Chinese Medicine Market of Yuzhou, China, and identified by Dr. Dongying Wang from College of Food Science and Technology, Henan University of Technology, Zhengzhou, China. Wheat flour (10 × 20.0 kg) manufactured by Yongxin Flour Co., Ltd., and sunflower oil (10 × 20.0 kg) manufactured by Dongsheng Oils and Fats Co., Ltd., were bought from Renrenle Supermarket. TBHQ, BHA, and BHT were purchased from Sigma. Moreover, all the chemicals employed were of analytical/HPLC grade and supplied by Senbo Biotechnology Co., Ltd.

### 2.2 Chemical analysis of NEO

First, NEO was extracted using our previous method (Wang et al., 2018, 2019). Briefly, 20.0 kg of nutmeg was smashed into particles (diameter <1.0 mm) by a grinder at 1 × 10^4^ r/min for 20 min (400Y, Boou Machinery Co., Ltd.), and these particles were averagely divided into 20 portions (each portion 1 kg). Each portion was put into a flask (4,000 ml) with 1,000 ml of water (w/v, 1:1), and a steam distillation apparatus (XH‐2000, Xinhu Experimental Device Co., Ltd.) was employed to distill NEO at 120°C for 6 hr. When the distillation process completed, the distillate was dumped into a separating funnel (500 ml) filled with 200 ml of ethyl acetate. In the partition process, the water phase was removed, while the organic phase was reserved. Subsequently, the organic phase for the distillate of the 20 portions was combined to obtain the crude NEO. After dried over anhydrous Na_2_SO_4_, the purified NEO was gained. Soon afterward, it was poured into a dark brown bottle and stored in a refrigerator (FYL‐YS‐73A; Fuyi Electric Co., Ltd.) at 4°C.

Second, the chemical analysis of NEO was carried out by the method of Piras et al. (2012). Briefly, operating at ionization energy of 70 eV, an Agilent 6890/5973 system installing a HP‐5MS capillary column (30.0 m × 0.25 mm × 0.25 μm, Agilent) was employed with He was the carrier gas at a flow rate of 1.1 ml/min. The oven temperature was programmed at 50°C and held for 2 min, and increased to 300°C by 10°C/min and held for 3 min. The temperatures of injector and detector were set at 250°C and 280°C, respectively. For injection, 1 μl of NEO was injected with a split of 20:1. In addition, the MS range (*m/z*) was set at 40–450, and the chemical constituents of NEO were elucidated by searching the NIST database 2018 (http://webbook.nist.gov.).

### 2.3 Selecting for the concentrations of antioxidants in frying

In order to obtain the optimal concentrations of antioxidants in vegetable oils during the frying process, the physical and chemical properties of vegetable oils containing the antioxidants should be measured after the frying process have been operating for at least 5 hr (Guo et al., 2016). According to Table 1, NEO, TBHQ, BHA, and BHT were directly added into sunflower oil at the concentrations of 0.04, 0.08, 0.12, 0.16 and 0.20 g/kg to prepare sunflower oil samples (each 10.0 kg), and a Control sample was prepared without any antioxidant as well. Before the frying process, all the sunflower oil samples were stored in brown bottles at 4°C. Herein, each sunflower oil sample was subjected to a frying process of 5 hr using a frying pan (L‐101C, Demashi Net Technology Co., Ltd.) at 180°C. For the frying process, the frying process began when the temperature of sunflower oil samples reached 180°C, and the acidity value (AV), iodine value (IV), peroxide value (PV), and *p*‐anisidine value (AnV) of all these sunflower oil samples were determined every hour in accordance with Chinese National Standards GB 5009.229–2016, GB/T 5532–2008, GB 5009.227–2016 and GB/T 24304–2009, respectively.

**Table 1:** Addition amount of antioxidants to sunflower oil samples for frying process

Antioxidant	Addition amount (antioxidant/sunflower oil, g/kg)					
	0	1	2	3	4	5
NEO	0	0.04	0.08	0.12	0.16	0.20
TBHQ	0	0.04	0.08	0.12	0.16	0.20
BHA	0	0.04	0.08	0.12	0.16	0.20
BHT	0	0.04	0.08	0.12	0.16	0.20

### 2.4 Deep‐frying of sunflower oil added by antioxidants

In the frying process above, the optimal concentrations of NEO, TBHQ, BHA, and BHT were found to be 0.12, 0.12, 0.08, and 0.08 g/kg, respectively. Therefore, the concentrations of NEO, TBHQ, BHA, and BHT used in the deep‐frying process were chosen as 0.12, 0.12, 0.08, and 0.08 g/kg, respectively. For deep‐frying of Chinese *Maye*, NEO, TBHQ, BHA, and BHT were directly added into sunflower oil to obtain sunflower oil samples (each 10.0 kg) NEO‐0.12, TBHQ‐0.12, BHA‐0.08, and BHT‐0.08, respectively. Meanwhile, one sunflower oil sample with no antioxidant was used as a Control sample. Before the deep‐frying process, all the sunflower oil samples were stored in brown bottles at 4°C, and 20.0 kg of wheat flour dough manufactured by a dough maker (HMJ‐A35A1, Bear Kitchen Appliances Co, Ltd.) was prepared to fry Chinese *Maye*. For the deep‐frying process, when the temperature of the sunflower oil samples reached 180°C, the deep‐frying process began and it was continued for 30 hr. During the deep‐frying process, the values for total polar compounds (TPC) and thiobarbituric acid (TBA) of the sunflower oil samples were measured every 6 hr in terms of Chinese National Standards GB/T 5009.202–2016 and GB 5009.181–2016, respectively. Furthermore, in terms of Chinese National Standard GB/T 22500–2008, the values for conjugated dienes (CD) and conjugated trienes (CT) were determined every 6 hr.

### 2.5 Sensory analysis for frying product, Chinese *Maye*

In the deep‐frying process, the evaluation for the sensory properties of Chinese *Maye* including flavor, taste, crispness, and overall acceptance was performed every 6 hr using a 10‐point hedonic scale (10 representing the highest intensity, while 1 representing the lowest intensity) by means of a trained panel. The panel was composed by 50 undergraduate students from Institute of Chinese Medicine Health Care, Guangdong Food and Drug Vocational College, Guangzhou, China, and 50 semitrained panelists from consumer representatives of Xinyuan Wholesale Food Market, Guangzhou, China, and all of them had sensory analysis in their curriculum and expressed an interest to undertake the work. Before the sensory analysis, they were trained in four 1‐hr sessions for the optimization and calibration of accuracy in interpretation and repeatability, and given enough time to familiarize themselves with the sensory analysis procedure. Moreover, the meaning of flavor, taste, crispness, and overall acceptance was explained to them to reduce their misunderstanding. In the evaluation session, the Chinese *Maye* were labeled with three‐digit codes at random, and panelists received a maximum of five samples to evaluation.

### 2.6 Deep‐frying of sunflower oil added by active compounds from NEO

First, in order to search for the active compounds with antioxidant effects in NEO, the fractionation guided by antioxidant activity was carried out using the dot‐blot test on TLC using silica gel Al plates (Adiani, Gupta, Chatterjee, Variyar, & Sharma, 2015). In brief, 10 μl of NEO was spotted on a piece of TLC plate, and then, the plate was eluted with the solvent system hexane‐ethyl acetate (80:20). After drying, the plate was sprayed with 160 μM of methanol solution of DPPH, and the bright yellow spots on the purple background of the sprayed plate was considered as active compounds with inhibitory effect against DPPH^•^. For the fractionation of them, preparative TLC plates produced with silica gel Al were used to with the same solvent system, and the active compounds with the corresponding Rf values were eluted with ether. Finally, the active compounds, limonene, terpinolene, and geranyl acetate were isolated, and their chemical structures were identified by HRESIMS and ^1^H‐NMR.

Second, for the evaluation of the antioxidant effects of the active compounds in the deep‐frying for Chinese *Maye*, limonene (0.12 g/kg × 14.5% = 17.4 mg/kg), terpinolene (0.12 g/kg × 8.4% = 10.1 mg/kg), and geranyl acetate (0.12 g/kg × 25.9% = 31.1 mg/kg) were added into sunflower oil at their corresponding concentrations to obtain sunflower oil samples (each 10 kg, Table 2) LI‐SFO, TE‐SFO and GE‐SFO, respectively, together with sunflower oil samples Control (nothing added, Control) and TBHQ (0.12 g/kg, TBHQ). Subsequently, the deep‐frying process was performed and continued for 30 hr, and the values for TPC, TBA, CD, and CT of sunflower oil samples were determined every 6 hr as well. Meanwhile, the sensory analysis of Chinese *Maye* was carried out.

**Table 2:** Chemical composition of NEO^a^

No.1	Kovats index^a^	Compound name	Molecular formula	w/w (%)
1	934	*α*‐Pinene	C_10_H_16_	1.5 ± 0.1
2	978	*β*‐Pinene	C_10_H_16_	2.8 ± 0.2
3	991	Myrcene	C_10_H_16_	2.1 ± 0.1
4	1010	*γ*‐Terpinene	C_10_H_16_	1.6 ± 0.1
5	1018	*α*‐Terpinene	C_10_H_16_	0.9 ± 0.1
6	1033	Limonene	C_10_H_16_	14.5 ± 1.2
7	1049	*trans*‐*β*‐Ocimene	C_10_H_16_	10.5 ± 0.9
8	1090	Terpinolene	C_10_H_16_	8.4 ± 0.6
9	1100	Linalool	C_10_H_18_O	1.2 ± 0.1
10	1182	Terpineol‐4	C_10_H_18_O	0.9 ± 0.1
11	1197	*β*‐Fenchyl alcohol	C_10_H_18_O	0.7 ± 0.1
12	1198	Caprylic acid	C_10_H_16_O_2_	12.6 ± 1.3
13	1291	Isobornyl acetate	C_12_H_20_O_2_	0.9 ± 0.1
14	1294	Safrole	C_10_H_10_O_2_	0.8 ± 0.1
15	1353	2,6‐Dimethyl 2,6‐octadiene	C_10_H_18_	0.5 ± 0.1
16	1363	Eugenol	C_10_H_12_O_2_	1.2 ± 0.1
17	1386	Geranyl acetate	C_12_H_20_O_2_	25.9 ± 2.9
18	1432	*trans*‐*β*‐Caryophyllene	C_15_H_24_	1.5 ± 0.2
19	1441	*trans*‐*α*‐Bergamotene	C_15_H_24_	1.4 ± 0.1
20	1459	*trans*‐*β*‐Farnesene	C_15_H_24_	1.4 ± 0.2
21	1506	*α*‐Muurolene	C_15_H_24_	1.0 ± 0.1
22	1529	Myristicin	C_11_H_12_O_3_	0.9 ± 0.1
23	1559	Elemicin	C_12_H_16_O_3_	0.8 ± 0.2
24	1608	Guaiol	C_15_H_26_O	0.7 ± 0.1
25	2362	Heptacosane	C_27_H_56_	1.5 ± 0.2
26	2640	Octadecane	C_18_H_38_	1.8 ± 0.2
Total			98.0 ± 8.6	

a Relative retention indices (RRI) on HP‐5MS column.

### 2.7 Statistical analysis

All of the experiments were carried out in duplicate. In the manuscript, unless otherwise indicated, the data are presented as mean values in test, and the data presented in figures and tables are presented as mean value ± standard deviations (*SD*). In statistical analysis, GraphPad Prism 6 (GraphPad Software) was employed to analyze the data by means of one‐way analysis of variance, where *p* < .05 was identified as statistically significant, and *p* < .01 was identified as and highly significant.

## 3 RESULTS AND DISCUSSION

### 3.1 Chemical constituents of NEO

In the past few years, the essential oil of nutmeg was famous for its attractive fragrance, so that it has been extracted using nutmeg from area to area by means of different methods, and its chemical constitution has been frequently investigated using GC‐MS (Bouchachia, Benkaci‐Ali, Eppe, & Scholl, 2017; da Rocha Voris et al., 2018). As shown in Table 2, 26 compounds were identified, representing 98.0% of the essential oil in this study. The extraction yield of the NEO was 6.4 g/kg of dried nutmeg. The major compounds were geranyl acetate (25.9 g/100 g), limonene (14.5 g/100 g), caprylic acid (12.6 g/100 g), and *trans*‐*β*‐ocimene (10.5 g/100 g). The extraction yield and chemical composition of NEO were both quite different from these previous studies, where the extraction yields were 2.5 – 5.0 g/kg of dried nutmeg, and the major compounds were considered to be sabinene (31.5–38.8 g/100 g), α‐pinene (12.7–14.1 g/100 g), and *β*‐pinene (10.0–12.1 g/100 g). The possible reason may be the different growing environments of the plant. As reported, the extraction yields and chemical compositions for the essential oils of plants were always significantly influenced by plenty of factors, including planting origin (genotype, variety), planting region (climate, soil, etc), or even the used extraction process (Wang et al., 2018, 2017).

### 3.2 Concentrations of the antioxidants for frying

**Figure 1:**
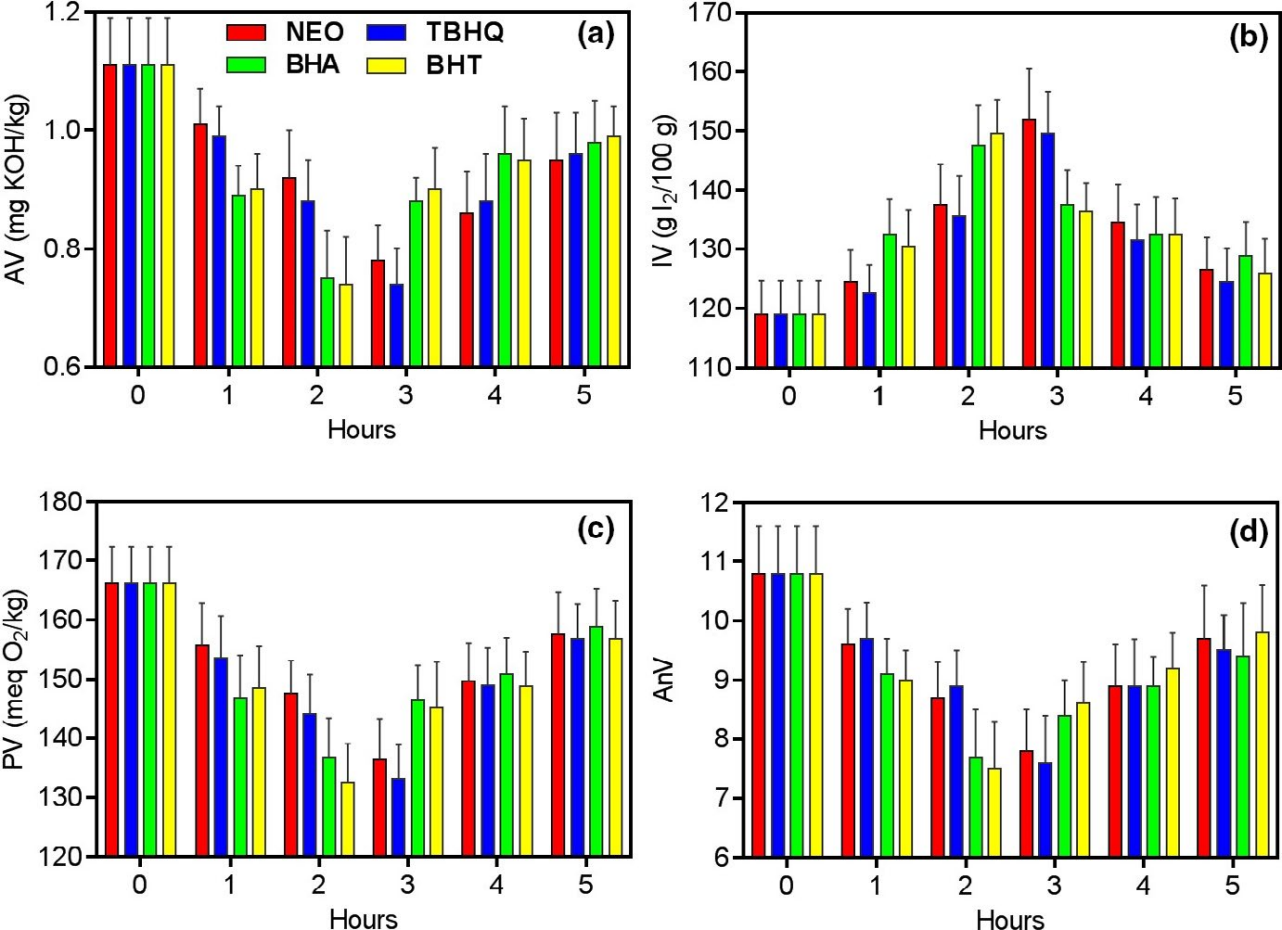
The influences of antioxidants on AV (a), IV (b), PV (c), and AnV (d) of sunflower oil samples during frying process at 180°C for 5 hr

Since the vegetable oils, frying temperature, or even frying pans that used during the frying process were different, the concentrations of antioxidants used in the frying process are also quite different, so that the selecting for concentrations of antioxidants is quite necessary (Bensmira, Jiang, Nsabimana, & Jian, 2007). As mentioned above, the physical–chemical properties of vegetable oils added by antioxidants during the frying process for at least 5 hr should be determined in details (Guo et al., 2016). As shown in Figure 1, during the frying process of 5 hr, all the administrations displayed two trends of evaluation. For NEO and TBHQ, the antioxidant effects increased at the concentrations 0.04–0.12 g/kg, while the antioxidant effects decreased at the concentrations 0.12–0.20 g/kg. For BHA and BHT, the antioxidant effects increased at the concentrations 0.04–0.08 g/kg, while the antioxidant effects decreased at the concentrations 0.08–0.20 g/kg. In other words, the relatively high antioxidant activity of NEO, TBHQ, BHA, and BHT were found when they were added at 0.12, 0.12, 0.08, and 0.08 g/kg, respectively. Therefore, this concentration of antioxidant was able to use to stabilize and retard frying oil oxidation, so that the concentrations of them used during the frying process were selected as 0.12, 0.12, 0.08, and 0.08 g/kg, respectively.

### 3.3 Influences of antioxidants on sunflower oil in deep‐frying

**Figure 2:**
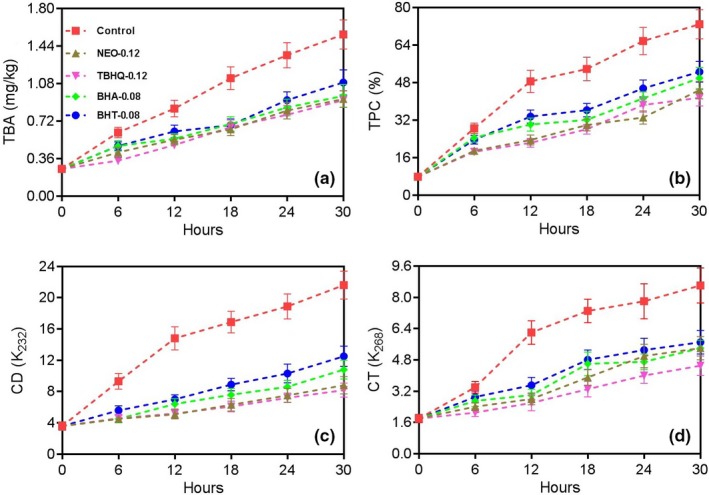
The influences of antioxidants on TBA (a), TPC (b), CD (c), and CT (d) of sunflower oil samples during the deep‐frying of Chinese *Maye* at 180°C for 30 hr

In the frying process, relating to the content of malondialdehyde (MDA) generated during the oxidation of vegetable oils, the TBA level is frequently employed as an indicator to measure the secondary products of them (Blasi et al., 2018; Montesano et al., 2019). TPC level is another indicator of much importance to evaluate the quality of vegetable oils. Since these substances of vegetable oils are not volatile and represent the major reaction products at frying temperature, to measure the TPC level offers us a reliable parameter for evaluating the oxidative stability of frying oils (Mattäus, Pudel, Chen, Achary, & Thiyam‐Holländer, 2014). The influence of antioxidant addition on the changes of TBA and TPC levels in sunflower oil samples during the deep‐frying process is shown in Figure 2. During the whole frying process, the TBA and TPC levels of the Control sample were prominently (*p* < .05) higher than that of the samples added by antioxidants, and the levels for TBA and TPC of sunflower oil samples improved with the time went on. Interestingly, the addition of antioxidants including NEO, TBHQ, BHA, and BHT exerted significant inhibitory effects against the improvement of them (*p* < .05), with the TBA and TPC levels at 30 hr of sunflower oil samples NEO‐0.12, TBHQ‐0.12, BHA‐0. 08, and BHT‐0.08 were 0.93, 0.92, 0.96, and 1.06 mg/kg, and 44.8%, 41.5%, 49.9%, and 52.6%, respectively, implying the relative order of their antioxidant effects was TBHQ at 0.12 g/kg > NEO at 0.12 g/kg > BHA at 0.08 g/kg > BHT at 0.08 g/kg. The results proved that NEO at 0.12 g/kg was able to inhibit the oxidation of sunflower oil during the deep‐frying process, and the possible reason of the phenomenon may be attributed to the natural compounds still existed in sunflower oil samples at the high temperature, 180°C, which could exhibit antioxidant effects (Blasi et al., 2018; Cardoso‐Ugarte, Morlán‐Palmas, & Sosa‐Morales, 2013; Montesano et al., 2019).

During the frying process, the oxidation of polyunsaturated fatty acids (PUFA) always leads to the generation of peroxides. After the formation of peroxides, the nonconjugated double bonds in PUFA undergoes a rearrangement, which results in the generation of CD (absorb at 232 nm, K_232_). Meanwhile, for PUFA containing three or more double bonds, the conjugation can be extended to contain another double bond which brings about the generation of CT (absorb at 268 nm, K_268_, Poiana, 2012; Redondo‐Cuevas, Castellano, & Raikos, 2017). Hence, CD and CT are parameters used for determining the amount of secondary products. The influence of antioxidant addition on the changes of CD and CT levels in sunflower oil samples during the deep‐frying process was shown in Figure 2. During the whole frying process, the CD and CT levels of the Control sample were obviously (*p* < .05) higher than that of the samples added by antioxidants, and the levels of CD and CT of sunflower oil samples improved with the time went on. Interestingly, the addition of antioxidants including NEO, TBHQ, BHA, and BHT exerted significant inhibitory effects against the improvement of them (*p* < .05), with the CD and CT levels at 30 hr of sunflower oil samples NEO‐0.12, TBHQ‐0.12, BHA‐0.08, and BHT‐0.08 were 8.8, 8.2, 10.8, and 12.5, and 5.4, 4.5, 5.5, and 5.7, respectively, implying the relative order of their antioxidant effects was TBHQ at 0.12 g/kg > NEO at 0.12 g/kg > BHA at 0.08 g/kg > BHT at 0.08 g/kg, too. The results also demonstrated that NEO at 0.12 g/kg was able to restrain the oxidation of sunflower oil during the deep‐frying process, which was in agreement with the results of TBA and TPC levels mentioned above. For the second time, the possible reason of the phenomenon may be the existence of some nature compounds with antioxidant effects, which still remained in the sunflower oil at 180°C (Blasi et al., 2018; Cardoso‐Ugarte et al., 2013; Montesano et al., 2019). The exploration herein is in agreement with the previous studies, and the antioxidative potential of the essential oil from nutmeg was confirmed again (Kapoor et al., 2013; Šojić et al., 2015).

### 3.4 Influences of antioxidants on sensory properties of Chinese *Maye*

As known, if we want to evaluate the suitability of vegetable oils used in the frying process, the most important is the sensory analysis of the fried products. There is no doubt that the consumer will refuse to use the vegetable oils if the fried products are not attractive to them. After all, during the frying process, a remarkable part of the frying medium, vegetable oils, is taken up by the fried products to the extent that the sensory properties of the vegetable oils are able to strongly influence the sensory properties of the fried products (Taha et al., 2014; Tohma & Turan, 2015). The influences of NEO sensory properties of Chinese *Maye* including flavor, taste, crispness, and overall acceptability were shown in Table 3. During the frying process of 30 hr, the sensory properties for Chinese *Maye* of all the sunflower oil samples were gradually dropped. Quite interestingly, compared with the Control sunflower oil sample, sunflower oil sample added by NEO at 0.12 g/kg had obviously high values of the sensory properties for Chinese *Maye* from 12 hr to 30 hr (*p* < .05 or *p* < .01), with the point 6.99, 6.29, 6.89, and 6.98 for its flavor, taste, crispness, and overall acceptability at 30 hr, respectively. Meanwhile, the additions of TBHQ at 0.12 g/kg, BHA at 0.08 g/kg, and BHT at 0.08 g/kg to sunflower oil could not markedly influence the sensory properties of Chinese *Maye* (*p* > .05), in relative to that of the Control sunflower oil sample. The results exhibited that the sensory properties of Chinese *Maye* including flavor, taste, crispness, and overall acceptability could be improved by the addition of NEO at 0.12 g/kg. In fact, nutmeg has long been employed as one spice in the frying process in China, such as *Maye* and *Youtiao* in Henan province, China (An et al., 2017). Herein, NEO was found to increase the sensory properties of Chinese *Maye*, so that the aroma components of nutmeg were attributed to its essential oil, and the suitability of sunflower oil added by NEO at 0.12 g/kg was demonstrated as well.

**Table 3:** The influences of NEO on flavor, taste, crispness, and overall acceptability of Chinese *Maye* a

Items	Hours	Control	NEO−0.12	TBHQ−0.12	BHA−0.08	BHT−0.08
Flavor	0	8.44 ± 0.66	8.44 ± 0.66	8.44 ± 0.66	8.44 ± 0.66	8.44 ± 0.66
	6	7.55 ± 0.55	8.17 ± 0.55	7.65 ± 0.44	7.89 ± 0.48	7.51 ± 0.52
	12	6.96 ± 0.47	7.86 ± 0.44^b^	6.93 ± 0.53	7.28 ± 0.56	6.86 ± 0.56
	18	6.32 ± 0.36	7.46 ± 0.45^c^	6.41 ± 0.54	6.84 ± 0.39	6.45 ± 0.39
	24	5.86 ± 0.39	7.25 ± 0.42^c^	5.99 ± 0.38	6.32 ± 0.61	5.94 ± 0.35
	30	5.11 ± 0.33	6.99 ± 0.34^c^	5.37 ± 0.58	5.65 ± 0.49	5.29 ± 0.46
Taste	0	8.67 ± 0.59	8.67 ± 0.59	8.67 ± 0.59	8.67 ± 0.59	8.67 ± 0.59
	6	7.48 ± 0.48	7.93 ± 0.59	7.62 ± 0.42	7.76 ± 0.56	7.51 ± 0.38
	12	6.39 ± 0.39	7.55 ± 0.39^b^	6.52 ± 0.52	6.68 ± 0.47	6.44 ± 0.29
	18	5.54 ± 0.31	7.01 ± 0.44^c^	5.71 ± 0.44	5.83 ± 0.55	5.60 ± 0.41
	24	4.69 ± 0.41	6.58 ± 0.66^c^	4.82 ± 0.39	4.92 ± 0.64	4.75 ± 0.43
	30	4.11 ± 0.32	6.29 ± 0.59^c^	4.28 ± 0.36	4.51 ± 0.39	4.19 ± 0.37
Crispness	0	8.96 ± 0.81	8.96 ± 0.81	8.96 ± 0.81	8.96 ± 0.81	8.96 ± 0.81
	6	8.14 ± 0.71	8.44 ± 0.59	8.26 ± 0.32	8.33 ± 0.58	8.10 ± 0.41
	12	7.36 ± 0.56	8.01 ± 0.68^b^	7.59 ± 0.54	7.78 ± 0.62	7.31 ± 0.39
	18	6.23 ± 0.57	7.78 ± 0.55^c^	6.49 ± 0.38	6.79 ± 0.49	6.31 ± 0.38
	24	5.32 ± 0.46	7.36 ± 0.61^c^	5.55 ± 0.35	5.83 ± 0.71	5.36 ± 0.31
	30	4.42 ± 0.38	6.89 ± 0.45^c^	4.68 ± 0.41	4.82 ± 0.48	4.61 ± 0.29
Overall acceptability	0	8.89 ± 0.76	8.89 ± 0.76	8.89 ± 0.76	8.89 ± 0.76	8.89 ± 0.76
	6	7.33 ± 0.61	8.31 ± 0.71^b^	7.48 ± 0.61	7.71 ± 0.81	7.38 ± 0.53
	12	6.39 ± 0.59	7.98 ± 0.49^c^	6.59 ± 0.52	6.58 ± 0.61	6.46 ± 0.58
	18	5.56 ± 0.45	7.46 ± 0.36^c^	5.74 ± 0.47	5.89 ± 0.55	5.69 ± 0.49
	24	5.03 ± 0.51	7.21 ± 0.49^c^	5.33 ± 0.58	5.45 ± 0.47	5.14 ± 0.60
	30	4.45 ± 0.48	6.98 ± 0.65^c^	4.87 ± 0.62	5.09 ± 0.53	4.55 ± 0.49

a Values are expressed as means ± *SD* (*n* = 100).

b As compared to control group at the same time: *p* < .05.

c As compared to control group at the same time: *p* < .01.

### 3.5 Influences of active compounds of NEO on sunflower oil and Chinese *Maye*

**Figure 3:**
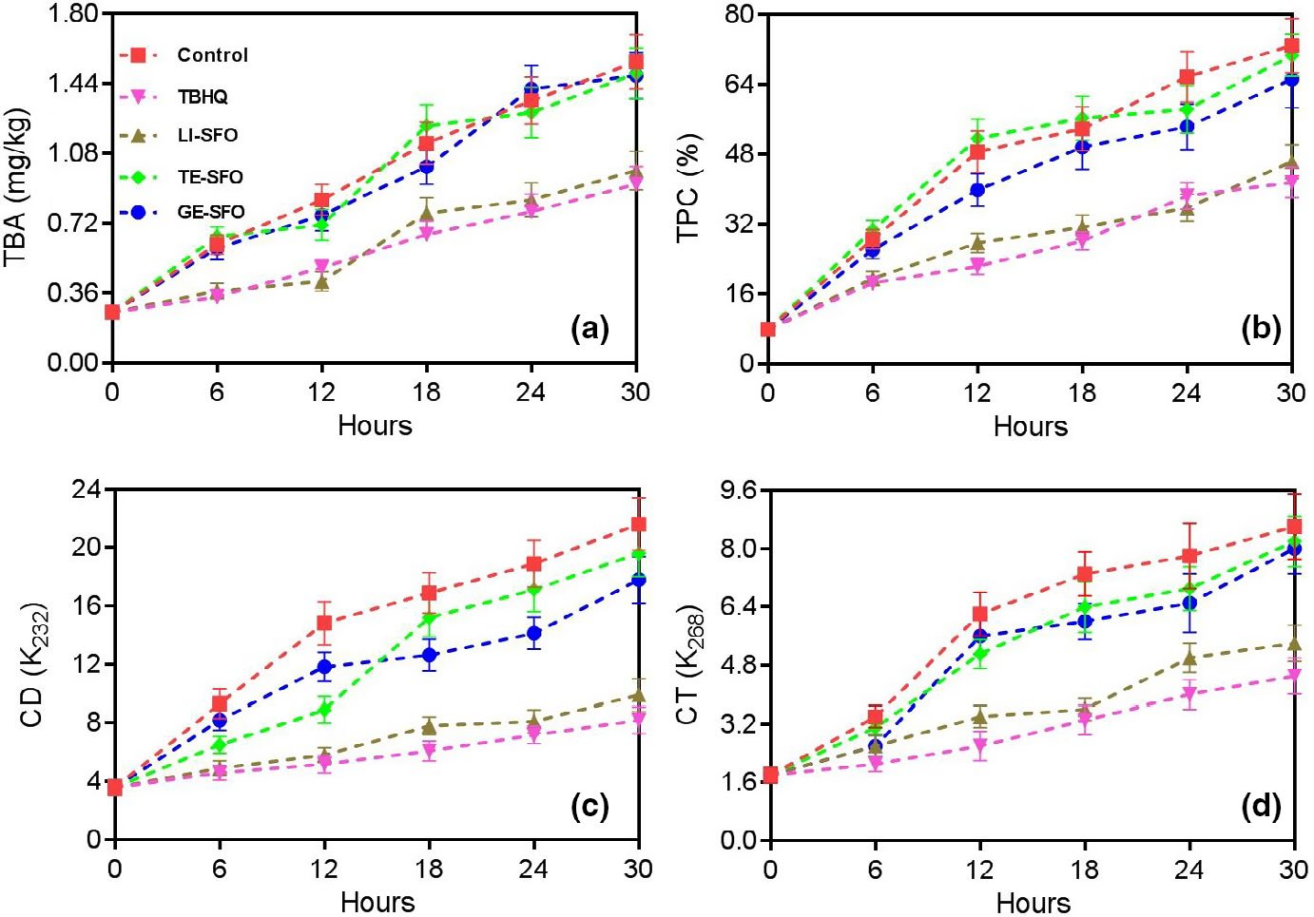
The influences of limonene, terpinolene, and geranyl acetate on TBA (a), TPC (b), CD (c), and CT (d) of sunflower oil samples during the deep‐frying of Chinese *Maye* at 180°C for 30 hr

The antioxidant activity‐guided fractionation of 5.0 NEO resulted in the isolation for three active compounds, 575.4 mg of limonene, 320.5 mg of terpinolene, and 1,002.1 mg of geranyl acetate, with their isolation yield 79.4%, 76.3%, and 77.4%, respectively. After the addition of limonene, terpinolene, and geranyl acetate at their corresponding contents determined by GC‐MS to sunflower oil, the sunflower oil samples were also used in the deep‐frying of Chinese *Maye*. Compared with the Control sunflower oil sample, the addition of limonene at 17.4 mg/kg to sunflower oil could dramatically inhibit the oxidation of sunflower oil during the deep‐frying process, with the values of TBA, TPC, CD, and CT (Figure 3d) were 0.99 mg/kg, 46.2%, 9.9, and 5.4 at 30 hr, respectively (Figure 3, *p* < .05). Moreover, in relative to that of the Control sunflower oil sample, the addition of limonene at 17.4 mg/kg to sunflower oil could also dramatically inhibit the drop of the sensory properties of Chinese *Maye* from 12–30 hr, with the point 7.03, 6.33, 6.93, and 7.06 for flavor, taste, crispness, and overall acceptability at 30 hr (Table 4). In the meantime, the other two active compounds, terpinolene and geranyl acetate, did not reveal antioxidant effect and sensory potentiation (*p* > .05). Consequently, the antioxidant effect and sensory potentiation of NEO were attributed to limonene. The results were in accordance with the previous studies, where limonene was demonstrated to reduce the frequency of micronucleus and DNA damage induced using H_2_O_2_, prevent early lipidemic‐oxidative stress‐induced detrimental changes, and normalize the cardiovascular disease risk parameters by means of its antioxidant activity (Ahmad & Beg, 2013; Bacanlı et al., 2015).

**Table 4:** The influences of active compounds on flavor, taste, crispness, and overall acceptability of Chinese *Maye* a

Items	Hours	Control	TBHQ−0.12	LI−17.4	TE−11.1	GE−31.1
Flavor	0	8.44 ± 0.66	8.44 ± 0.66	8.44 ± 0.66	8.44 ± 0.66	8.44 ± 0.66
	6	7.55 ± 0.55	7.65 ± 0.44	8.14 ± 0.54	7.84 ± 0.44	7.58 ± 0.45
	12	6.96 ± 0.47	6.93 ± 0.53	7.91 ± 0.63^b^	7.21 ± 0.68	6.66 ± 0.62
	18	6.32 ± 0.36	6.41 ± 0.54	7.51 ± 0.47^c^	6.80 ± 0.42	6.41 ± 0.33
	24	5.86 ± 0.39	5.99 ± 0.38	7.32 ± 0.44^c^	6.36 ± 0.33	5.99 ± 0.39
	30	5.11 ± 0.33	5.37 ± 0.58	7.03 ± 0.52^c^	5.69 ± 0.56	5.35 ± 0.41
Taste	0	8.67 ± 0.59	8.67 ± 0.59	8.67 ± 0.59	8.67 ± 0.59	8.67 ± 0.59
	6	7.48 ± 0.48	7.62 ± 0.42	7.97 ± 0.57	7.71 ± 0.65	7.57 ± 0.44
	12	6.39 ± 0.39	6.52 ± 0.52	7.60 ± 0.41^b^	6.64 ± 0.45	6.37 ± 0.32
	18	5.54 ± 0.31	5.71 ± 0.44	7.04 ± 0.46^c^	5.88 ± 0.66	5.68 ± 0.39
	24	4.69 ± 0.41	4.82 ± 0.39	6.62 ± 0.59^c^	4.86 ± 0.35	4.71 ± 0.52
	30	4.11 ± 0.32	4.28 ± 0.36	6.33 ± 0.54^c^	4.58 ± 0.47	4.22 ± 0.45
Crispness	0	8.96 ± 0.81	8.96 ± 0.81	8.96 ± 0.81	8.96 ± 0.81	8.96 ± 0.81
	6	8.14 ± 0.71	8.26 ± 0.32	8.46 ± 0.53	8.38 ± 0.63	8.17 ± 0.47
	12	7.36 ± 0.56	7.59 ± 0.54	8.07 ± 0.72^b^	7.71 ± 0.55	7.38 ± 0.42
	18	6.23 ± 0.57	6.49 ± 0.38	7.81 ± 0.58^c^	6.72 ± 0.53	6.47 ± 0.38
	24	5.32 ± 0.46	5.55 ± 0.35	7.43 ± 0.44^c^	5.88 ± 0.47	5.28 ± 0.33
	30	4.42 ± 0.38	4.68 ± 0.41	6.93 ± 0.41^c^	4.89 ± 0.44	4.79 ± 0.38
Overall acceptability	0	8.89 ± 0.76	8.89 ± 0.76	8.89 ± 0.76	8.89 ± 0.76	8.89 ± 0.76
	6	7.33 ± 0.61	7.48 ± 0.61	8.35 ± 0.57^b^	7.78 ± 0.76	7.44 ± 0.66
	12	6.39 ± 0.59	6.59 ± 0.52	8.03 ± 0.52^c^	6.50 ± 0.55	6.41 ± 0.47
	18	5.56 ± 0.45	5.74 ± 0.47	7.50 ± 0.63^c^	5.81 ± 0.65	5.72 ± 0.54
	24	5.03 ± 0.51	5.33 ± 0.58	7.26 ± 0.46^c^	5.49 ± 0.61	5.33 ± 0.42
	30	4.45 ± 0.48	4.87 ± 0.62	7.06 ± 0.64^c^	5.17 ± 0.58	4.62 ± 0.44

a Values are expressed as means ± *SD* (*n* = 100).

LI‐17.4: limonene at 17.4 mg/kg, TE‐10.1: terpinolene at 10.1 mg/kg; GE‐31.1 geranyl acetate at 31.1 mg/kg.

b As compared to control group at the same time: *p* < .05.

c As compared to control group at the same time: *p* < .01.

## 4 CONCLUSION

In a word, NEO at 0.12 g/kg was able to not only prominently improve the oxidative stability of sunflower oil during the deep‐frying process at 180°C for 30 hr, but also prominently increase the sensory properties including flavor, taste, crispness, and overall acceptability of fried product, *Chinese Maye*. Meanwhile, although the synthetic antioxidants such as TBHQ, BHA, and BHT could also exhibit observably antioxidant effects during the deep‐frying process with the relative order: TBHQ at 0.12 g/kg > NEO at 0.12 g/kg > BHA at 0.08 g/kg > BHT at 0.08 g/kg, they could not ameliorate the sensory properties of Chinese *Maye*. Furthermore, the antioxidant effect and sensory improvement of the essential oil were attributed to limonene. Now, the function mechanism of limonene in the deep‐frying process and the toxicology of the sunflower oil added by limonene at 17.1 mg/kg are being explored in our laboratory. Once these experiments finished, limonene will be used to substitute the synthetic antioxidants in the deep‐frying process, and the sunflower oil flavored by NEO can be developed as flavored oils for deep‐frying.

## CONFLICT OF INTEREST

None.

## ETHICAL APPROVAL

This study does not involve any human or animal testing.

## References

[fsn31333-bib-0001] Abourashed, E. A. , & El‐Alfy, A. T. (2016). Chemical diversity and pharmacological significance of the secondary metabolites of nutmeg (*Myristica fragrans* Houtt.). Phytochemistry Review, 15, 1035–1056. 10.1007/s11101-016-9496-x PMC5222521 28082856

[fsn31333-bib-0002] Acuña, U. M. , Carcache, P. J. B. , Matthew, S. , & de Blanco, E. J. C. (2016). New acyclic bis phenylpropanoid and neolignans, from *Myristica fragrans* Houtt., exhibiting PARP‐1 and NF‐jB inhibitory effects. Food Chemistry, 202, 269–275. 10.1016/j.foodchem.2016.01.060 26920294 PMC4770456

[fsn31333-bib-0003] Adiani, V. , Gupta, S. , Chatterjee, S. , Variyar, P. S. , & Sharma, A. (2015). Activity guided characterization of antioxidant components from essential oil of Nutmeg (*Myristica fragrans*). Journal of Food Science and Technology, 52, 221–230. 10.1007/s13197-013-1034-7.

[fsn31333-bib-0004] Ahmad, S. , & Beg, Z. H. (2013). Hypolipidemic and antioxidant activities of thymoquinone and limonene in atherogenic suspension fed rats. Food Chemistry, 138, 1116–1124. 10.1016/j.foodchem.2012.11.109 23411222

[fsn31333-bib-0005] Aladedunye, F. , & Matthäus, B. (2014). Phenolic extracts from *Sorbus aucuparia* (L.) and *Malus baccata* (L.) berries: Antioxidant activity and performance in rapeseed oil during frying and storage. Food Chemistry, 159, 273–281. 10.1016/j.foodchem.2014.02.139 24767055

[fsn31333-bib-0006] An, K. J. , Liu, Y. L. , & Liu, H. L. (2017). Relationship between total polar components and polycyclic aromatic hydrocarbons in fried edible oil. Food Additives & Contaminants: Part A, 34, 1596–1605. 10.1080/19440049.2017.1338835 28590158

[fsn31333-bib-0007] Bacanlı, M. , Başaran, A. A. , & Başaran, N. (2015). The antioxidant and antigenotoxic properties of citrus phenolics limonene and naringin. Food and Chemical Toxicology, 81, 160–170. 10.1016/j.fct.2015.04.015 25896273

[fsn31333-bib-0008] Bensmira, M. , Jiang, B. , Nsabimana, C. , & Jian, T. (2007). Effect of lavender and thyme incorporation in sunflower seed oil on its resistance to frying temperatures. Food Research International, 40, 341–346. 10.1016/j.foodres.2006.10.004

[fsn31333-bib-0009] Blasi, F. , Rocchetti, G. , Montesano, D. , Lucini, L. , Chiodelli, G. , Ghisoni, S. , … Cossignani, L. (2018). Changes in extra‐virgin olive oil added with *Lycium barbarum* L. carotenoids during frying: Chemical analyses and metabolomic approach. Food Research International, 105, 507–516. 10.1016/j.foodres.2017.11.061 29433242

[fsn31333-bib-0010] Bouchachia, C. , Benkaci‐Ali, F. , Eppe, G. , & Scholl, G. (2017). Effect of different parameters on composition of volatile components of *Myristica fragrans* seeds extracted by hydrodistillation assisted by microwave and head‐space solid‐phase micro‐extraction. Journal of Essential Oil Research, 29, 481–493. 10.1080/10412905.2017.1322006

[fsn31333-bib-0011] Cardoso‐Ugarte, G. A. , Morlán‐Palmas, C. C. , & Sosa‐Morales, M. E. (2013). Effect of the addition of basil essential oil on the degradation of palm olein during repeated deep frying of french fries. Journal of Food Science, 78, C978–C984. 10.1111/1750-3841.12166 23772857

[fsn31333-bib-0012] Chirinos, R. , Huamán, M. , Betalleluz‐Pallardel, I. , Pedreschi, R. , & Campos, D. (2011). Characterisation of phenolic compounds of Inca muña (*Clinopodium bolivianum*) leaves and the feasibility of their application to improve the oxidative stability of soybean oil during frying. Food Chemistry, 128, 711–716. 10.1016/j.foodchem.2011.03.093

[fsn31333-bib-0013] Gomes da Rocha Voris, D. , dos Santos Dias, L. , Alencar Lima, J. , dos Santos Cople Lima, K. , Pereira Lima, J. B. , & dos Santos Lima, A. L. (2018). Evaluation of larvicidal, adulticidal, and anticholinesterase activities of essential oils of *Illicium verum* Hook. f., *Pimenta dioica* (L.) Merr., and *Myristica fragrans* Houtt. against Zika virus vectors. Environmental Science and Pollution Research, 25, 22541–22551. 10.1007/s11356-018-2362-y 29808407

[fsn31333-bib-0014] Guo, Q. , Gao, S. , Sun, Y. , Gao, Y. , Wang, X. , & Zhang, Z. (2016). Antioxidant efficacy of rosemary ethanol extract in palm oil during frying and accelerated storage. Industrial Crops and Products, 94, 82–88. 10.1016/j.indcrop.2016.08.032

[fsn31333-bib-0015] Hwang, H.‐S. , Winkler‐Moser, J. K. , & Liu, S. X. (2012). Structural effect of lignans and sesamol on polymerization of soybean oil at frying temperature. Journal of the American Oil Chemists' Society, 89, 1067–1076. 10.1007/s11746-011-1994-6

[fsn31333-bib-0016] Jiménez, P. , García, P. , Bustamante, A. , Barriga, A. , & Robert, P. (2017). Thermal stability of oils added with avocado (*Persea americana* cv. Hass) or olive (*Olea europaea* cv. Arbequina) leaf extracts during the French potatoes frying. Food Chemistry, 221, 123–129. 10.1016/j.foodchem.2016.10.051 27979083

[fsn31333-bib-0017] Kapoor, I. P. S. , Singh, B. , Singh, G. , De Heluani, C. S. , De Lampasona, M. P. , & Catalan, C. A. N. (2013). Chemical composition and antioxidant activity of essential oil and oleoresins of nutmeg (*Myristica fragrans* houtt.) fruits. International Journal of Food Properties, 16, 1059–1070. 10.1080/10942912.2011.576357

[fsn31333-bib-0018] Mattäus, B. , Pudel, F. , Chen, Y. , Achary, A. , & Thiyam‐Holländer, U. (2014). Impact of Canolol‐enriched extract from heat‐treated canola meal to enhance oil quality parameters in deep‐frying: A comparison with rosemary extract and TBHQ‐fortified oil systems. Journal of the American Oil Chemists’ Society, 91, 2065–2076. 10.1007/s11746-014-2561-8

[fsn31333-bib-0019] Montesano, D. , Rocchetti, G. , Cossignani, L. , Senizza, B. , Pollini, L. , Lucini, L. , & Blasi, F. (2019). Untargeted metabolomics to evaluate the stability of extra‐virgin olive oil with added *Lycium barbarum* carotenoids during storage. Foods, 8, 179 10.3390/foods8060179 PMC6616970 31141920

[fsn31333-bib-0020] Piras, A. , Rosa, A. , Marongiu, B. , Atzeri, A. , Dessì, M. A. , Falconieri, D. , & Porcedda, S. (2012). Extraction and separation of volatile and fixed oils from seeds of *Myristica fragrans* by supercritical CO_2_: Chemical composition and cytotoxic activity on Caco‐2 cancer cells. Journal of Food Science, 77, C448–C453. 10.1111/j.1750-3841.2012.02618.x 22429024

[fsn31333-bib-0021] Poiana, M. A. (2012). Enhancing oxidative stability of sunflower oil during convective and microwave heating using grape seed extract. International Journal of Molecular Sciences, 13, 9240–9259. 10.3390/ijms13079240 22942764 PMC3430295

[fsn31333-bib-0022] Redondo‐Cuevas, L. , Castellano, G. , & Raikos, V. (2017). Natural antioxidants from herbs and spices improve the oxidative stability and frying performance of vegetable oils. International Journal of Food Science and Technology, 52, 2422–2428. 10.1111/ijfs.13526

[fsn31333-bib-0023] Šojić, B. , Tomović, V. , Kocić‐Tanackov, S. , Škaljac, S. , Ikonić, P. , Džinić, N. , … Kravić, S. (2015). Effect of nutmeg (*Myristica fragrans*) essential oil on the oxidative and microbial stability of cooked sausage during refrigerated storage. Food Control, 54, 282–286. 10.1016/j.foodcont.2015.02.007

[fsn31333-bib-0024] Su, L. , Yin, J. J. , Charles, D. , Zhou, K. , Moore, J. , & Yu, J. (2007). Total phenolic contents, chelating capacities, and radical‐scavenging properties of black peppercorn, nutmeg, rosehip, cinnamon and oregano leaf. Food Chemistry, 100, 990–997. 10.1016/j.foodchem.2005.10.058

[fsn31333-bib-0025] Taha, E. , Abouelhawa, S. , El‐Geddawy, M. , Sorour, M. , Aladedunye, F. , & Matthäus, B. (2014). Stabilization of refined rapeseed oil during deep‐fat frying by selected herbs. European Journal of Lipid Science and Technology, 116, 771–779. 10.1002/ejlt.201300279

[fsn31333-bib-0026] Tohma, S. , & Turan, S. (2015). Rosemary plant (*Rosmarinus officinalis* L.), solvent extract and essential oil can be used to extend the usage life of hazelnut oil during deep frying. European Journal of Lipid Science and Technology, 117, 1798–1990. 10.1002/ejlt.201400382

[fsn31333-bib-0027] Wang, D. , Fan, W. , Guan, Y. , Huang, H. , Yi, T. , & Ji, J. (2018). Oxidative stability of sunflower oil flavored by essential oil from *Coriandrum sativum* L. during accelerated storage. LWT ‐ Food Science and Technology, 98, 268–275. 10.1016/j.lwt.2018.08.055

[fsn31333-bib-0028] Wang, D. , Meng, Y. , Zhao, X. , Fan, W. , Yi, T. , & Wang, X. (2019). Sunflower oil flavored by essential oil from *Punica granatum cv. Heyinshiliu* peels improved its oxidative stability and sensory properties. LWT ‐ Food Science and Technology, 111, 55–61. 10.1016/j.lwt.2019.05.005

[fsn31333-bib-0029] Wang, L. , Liu, H. M. , & Qin, G. Y. (2017). Structure characterization and antioxidant activity of polysaccharides from Chinese quince seed meal. Food Chemistry, 234, 314–322. 10.1016/j.foodchem.2017.05.002 28551241

[fsn31333-bib-0030] Zhao, X. , Wu, H. , Wei, J. , & Yang, M. (2019). Quantification and characterization of volatile constituents in *Myristica fragrans* Houtt. by gas chromatography‐mass spectrometry and gas chromatography quadrupole‐time‐of‐flight mass spectrometry. Industrial Crops and Products, 130, 137–145. 10.1016/j.indcrop.2018.12.064

